# Reframing aging in dermatology: The role of the dermatologist in healthy aging

**DOI:** 10.1016/j.ijwd.2021.08.014

**Published:** 2021-09-03

**Authors:** Shreya A. Sreekantaswamy, Daniel C. Butler, Amit A. Shah

**Affiliations:** a Department of Dermatology, University of California, San Francisco, California; b University of Utah, School of Medicine, Salt Lake City, Utah; c Division of Community Internal Medicine, Mayo Clinic, Scottsdale, Arizona

**Keywords:** Aging, healthy aging, dermatology, geriatric dermatology, aging misconceptions, cutaneous aging

## Abstract

Dermatology is often tasked with balancing the clinical appearance of aging skin with the reality of what healthy aging means. In this article, we review some of the core principles of healthy aging and explore common misconceptions, both from patients and physicians, regarding aging. Recognition of the basics of healthy aging and awareness of these aging myths can empower providers to advise patients accurately and productively regarding their aging goals.

 

**What is known about this subject in regard to women and their families?**

• The media disproportionately targets women with anti-aging skin care advertisements, prompting many to approach dermatologists with requests for treatments to look younger.
• Such portrayal of dermatology in the media can also lead dermatologists to make assumptions about a patient's skin care goals based on sex, age, functional status, and overall appearance.
• Although the concept of healthy aging is important for both men and women, women often live longer than men and spend more years of their lives with functional limitations, making the maintenance of healthy aging of particular importance for the female gender.
• **What is new from this article as messages for women and their families?**
• A frank discussion is provided on several aging myths from the perspective of both the patient and the provider, with tips on how to navigate complex discussions regarding healthy aging and aging skin.

## Introduction

Ms. A is a 60-year-old woman who presents to the clinic. She wants to know what she should do to look younger. Examination reveals rhytids and age-related volume loss. The patient eats a healthy and well-balanced diet but lives a relatively sedentary lifestyle and has smoked one pack of cigarettes per day for 15 years. What would you recommend?

This is a common scenario for the dermatologist, who is often challenged with balancing the clinical appearance of undesired skin aging with the role of a critical health care provider in the aging sphere who must acknowledge the inevitability of becoming older. Having a foundational understanding of the reality of healthy aging can help dermatologists navigate these sometimes conflicting responsibilities, empowering providers to advise patients accurately and productively. We will revisit Ms. A and the appropriate recommendations for her later in this article.

## What is healthy aging?

Geriatricians are the experts at the medicine of aging. This includes a clinical focus on the diseases of aging and expert knowledge of healthy aging. These topics are so vast that geriatricians rely on collaboration with other specialties, such as dermatology, to strengthen these discussions with patients. This highlights the importance of collaboration to answer questions on aging.

Rather than simply being about living longer or looking younger, healthy aging is about increasing a healthy lifespan (i.e., years lived with a good quality of life and reduced morbidity). Research has shown that only 20% to 30% of an average person's lifespan can be attributed to genetics, and the main contributors to healthy aging are diet and lifestyle. Healthy behaviors may prevent detrimental epigenetic changes, such as telomere shortening and a reduction in immune function (Arsenis et al., 2017). Aging is therefore not a disease to be cured, but rather a process that can be optimized. Maintenance of healthy aging is of particular importance for women, given that they often live longer than men and spend more years of their lives with functional limitations (Carmel, 2019).

At this time, it is not possible to change an individual's longevity-associated genes, so what other factors can be optimized? An investigation of long-lived societies can provide some suggestions. There are five longevity hotspots around the world that appear to have optimized the aging process, resulting in long lives with high functional ability. Collectively, these hotspots are known as the blue ones. The blue zones project started in 2004 with a National Geographic expedition to find the secrets of certain societies leading long, healthy lives (Buettner and Skemp, 2016). Five communities were found where individuals were generally healthy and reached age 100 years at 10 times the rate of individuals in the United States. These blue zones are Loma Linda, California; Nicoya, Costa Rica; Sardinia, Italy; Ikaria, Greece; and Okinawa, Japan. Despite being located in different parts of the world with different lifestyles and practices (and genetics of their populations), the common lessons learned from these communities can be summed into nine points (Buettner and Skemp, 2016):

• Engage in moderate, regular physical activity
• Have a sense of purpose
• Reduce stress
• Stop eating when 80% full
• Consume a plant-based diet with limited meat intake
• Have a moderate red wine intake
• Feel a sense of societal connectedness
• Put family first
• Engage in spirituality or religion

Physicians are often considered by the public to be part of the antiaging treatment sphere; thus, dermatologists can learn from the literature on healthy aging, similar to what is seen in the blue zones, to navigate complex but common discussions about aging. Dermatologists are therefore in a unique position to be able to encourage patients to enact positive lifestyle changes. For example, the dermatologist caring for Ms. A in the opening case scenario should use the patient's concern about aging in the encounter as an opportunity to discuss smoking cessation. Linking the cutaneous changes she is worried about with her lifestyle habits could be instrumental in helping her stop smoking, which would not only improve her skin but also make a significant impact on her future morbidity and mortality. On further questioning, a provider might also find that Ms. A lives alone and is socially isolated with few friends outside of her workplace. Encouraging her to increase her social connectedness could also help her age healthily.

The process of healthy aging is different for every person, and assumptions should not be made based on age, functional ability, or appearance. There are, however, several key areas in which positive lifestyle change can improve skin health, which are discussed herein. Although skin aging is also affected by intrinsic factors, such as sex hormones and facial anatomy, which pertain to differences in aging between men and women, regular modification of these internal factors is not a part of the healthy aging discussion and will not be reviewed in this paper.

### Smoking

Cigarette smoke is associated with significant morbidity and mortality. Although smoking is more prevalent among men than women, women who are heavy smokers have been found to have a higher risk for earlier age of death and stroke than their male counterparts (Haghani et al., 2020). Smoking is also a well-known independent risk factor for premature skin aging. Cutaneous aging secondary to smoking characteristically manifests as a “smoker's face,” with prominent wrinkles, skin atrophy, and a gray complexion. This accelerated aging process has been demonstrated in an identical twin study and attributed to cigarette smoke's induction of free radicals and its effect in altering functions of cellular repair, defense, and extracellular matrix turnover by elevating levels of dermal matrix metalloproteinase-8 (Ortiz and Grando, 2012). Cigarette smoke is also known to delay wound healing and disrupt homeostasis of the epidermal barrier, with greater use of cigarettes found to negatively correlate with the barrier recovery rate (Xin et al., 2016).

### Physical activity

Routine physical activity has numerous health benefits. Individuals who meet or exceed the international recommendations for 150 minutes per week of moderate to vigorous physical activity have been found to have a 20% to 30% risk reduction for premature mortality and development of at least 25 chronic medical conditions (Warburton and Bredin, 2017). Exercise also benefits skin health by increasing blood flow, which is critical for the delivery of oxygen and nutrients to the skin. This in turn promotes collagen production that is essential for the formation of new skin cells. Although age leads to thickening of the stratum corneum and thinning of the dermis, Crane et al. (2015) found that men and women age >40 years who participated in ≥4 hours per week of high-intensity aerobic exercise had a thinner stratum corneum and a thicker dermis than individuals who participated in ≤1 hour of exercise per week. Furthermore, when a subset of sedentary older adults was enrolled in a 3-month cycling program, the researchers found a significant decrease in stratum corneum thickness and an increase in the collagen content of the reticular dermis when comparing pre- and postintervention skin samples from the participants (Crane et al., 2015).

The benefits of physical activity are not just limited to those who engage in 150 minutes of exercise per week. Studies have shown that clinically relevant health benefits (e.g., reduced risk for cardiovascular disease or type 2 diabetes) can be achieved even in patients who engage in physical activity <150 minutes per week, indicating that messaging that states that individuals must meet the standard amount of physical activity set by guidelines to benefit their health is misguided (Warburton and Bredin, 2017). In fact, most studies reveal that the relationship between health status and physical activity is nonlinear, and the greatest benefit is obtained in those who have a slight increase in activity from a previously sedentary lifestyle (Warburton and Bredin, 2017). Exercise is also known to make patients simply feel better, resulting in significant improvement in quality of life, especially in older adults (Awick et al., 2017).

### Healthy diet

Medicine has long championed the importance of balanced nutrition. Not only is a healthy diet integral to preventing chronic diseases, but improvements in diet have been shown to reduce cutaneous signs of aging. One example of a key relationship between diet and skin health is vitamin C, which is critical for collagen synthesis. Data from a study of 4025 women in the National Health and Nutrition Examination Survey cohort showed that higher vitamin C intake was associated with a lower likelihood of wrinkles, and a 17 g increase in fat and 50 g increase in carbohydrate intake were both associated with an increased likelihood of wrinkles (Cosgrove et al., 2007). In a longitudinal cohort study in Australia, adults age >45 years who ate foods high in antioxidants were found to have 10% less photoaging (assessed via silicone skin surface replicas) over the 15-year period of the study compared with those who did not (Hughes et al., 2021). Although various dietary regimens (e.g., Mediterranean, vegetarian) have been studied and touted for their health benefits over the years, what is most important is balanced nutrition with a reduced intake of red meat, long-term consumption of which has been linked to overall mortality, cardiovascular disease, colorectal cancer, and type 2 diabetes (Battaglia Richi et al., 2015).

### Stress

Stress results in the activation of the hypothalamic–pituitary–adrenal axis, leading to the production of cortisol. Repeated and prolonged cortisol exposure in chronic stress can lead to cortisol dysfunction, ultimately resulting in reduced immune function, impaired wound healing, and increased inflammation, which can exacerbate inflammatory dermatologic conditions (Chen and Lyga, 2014). Although the exact impact of stress on skin aging is yet to be elucidated, cortisol has been found to lead to DNA damage (Flint et al., 2007), and stress-associated catecholamine stimulation has also been observed to contribute to DNA damage by degrading p53 (Hara et al., 2011), both of which can contribute to skin aging. Additionally, stress has been hypothesized to contribute to skin aging by resulting in the buildup of reactive oxygen species and telomere shortening (Chen and Lyga, 2014), which is especially detrimental for the skin given the frequency of skin cell reproduction (Aubert and Lansdorp, 2008). Apart from affecting the skin's intrinsic aging, from a cosmetic standpoint, signs of chronic stress often manifest as increased wrinkles and stress lines. Stress reduction could therefore potentially play a role in decreasing signs of cutaneous aging.

## Dermatology and aging

Dermatologists deal with aging skin on a daily basis. Although one patient might be concerned with how their skin is changing as they become older, another might be subject to cutaneous carcinoma resulting from years of sun exposure. With both patients, the dermatologist is tasked with navigating the blurry lines of what it means to have “aging skin.” Sometimes, effective communication on aging can be challenged by the different expectations of patients and providers. Owing to the media's portrayal of dermatology as a specialty centered around skin care and antiaging, society has been led to believe that the sole role of the dermatologist is to protect patients from displaying outward signs of aging. Yet, not only does this grossly oversimplify the complex medical conditions that dermatologists manage, it also misrepresents the role of the dermatologist in the aging sphere. Although dermatologists can ameliorate some cutaneous changes of age, it is not possible to reverse the process of aging in and of itself. In addition, dermatologists often carry their own biases toward patients, thinking, for example, that a patient in their 80s might care less about their appearance than someone in their 40s, even though the opposite might be the case. Therefore, it is the responsibility of dermatologists to advocate for evidence-based practice in patient interactions and avoid assumptions of where patients are in the aging process or aging-related skin goals. Herein, we explore several aging myths in dermatology and provide guidance on how to have a more in-depth discussion about healthy aging with patients.

## Aging misconceptions

### Myths from the patient's perspective

Myth 1: The dermatologist can make me younger.

When meeting patients who want to reverse the signs of cutaneous aging, a frank and open discussion on their goals and the capabilities of a dermatologist is important. Reasonable procedures and cosmeceuticals can be recommended, but it should be emphasized that despite reducing some of the signs associated with skin aging, dermatologists will never be able to change the inevitability of the aging process. Discussion should include lifestyle factors that can influence cutaneous aging, such as diet, exercise, and smoking habits (Battaglia Richi et al., 2015; Ortiz and Grando, 2012; Warburton and Bredin, 2017).

Myth 2: There is a simple cream or procedure that can help completely reverse the signs of aging.

Antiaging skin care advertisements can make it seem as though there is a simple, cure-all solution to aging. The reality is that even the best of procedures and cosmeceuticals will only have a temporary effect; often, multiple management strategies might be needed to achieve the desired appearance. Aging is complex and there is never a fix-all solution. While having a thorough discussion on the cosmetic options available, equal importance should be given to what healthy aging means for that patient. The diverse skillset and knowledge base of dermatologists give them a unique ability to address these specific concerns while broadening the discussion to general health recommendations.

### Myths and tips for the dermatologist

Myth for the older patient with functional decline: This patient no longer cares about appearance and is only interested in procedures related to improving functional abilities.

Tips to work with this patient: do not assume that a patient no longer cares about their appearance based on their age or functional status. Ask the patient what their goals of care are for the visit and, if warranted, engage in a conversation about options to meet those goals in a safe, goal-oriented way. One example is the consideration of Mohs surgery for a keratinocyte carcinoma of the face, assuming that an older adult would not care about the resulting postsurgical scar, which may lead to discordance between patient and provider.

Myth for the patient who lives alone with signs of poor self-care and no acute skin issues: The dermatologist no longer has a role in caring for this patient because there are no active skin concerns.

Tips to work with this patient: Although the patient might not have any acute skin concerns, dermatologists continue to be a valuable part of the medical team, observing clues suggestive of critical health factors. These observations may help a medical team understand and guide a patient with suggestions and referrals. For example, the long toenail sign, which refers to suboptimal overall foot hygiene, including uncut toenails, can be used by dermatologists to assess a patient's ability for self-care, providing valuable insight into their overall functional status (James et al., 2021). Additionally, every interaction with the health care system serves as an opportunity to encourage patients to continue positive lifestyle habits.

## Conclusion

Dermatologists are in a unique position to not only provide temporary cosmetic fixes for signs of cutaneous aging, but to address other factors that fundamentally affect the complex process of healthy aging. By embracing these conversations and connecting healthy aging principles with skin health, dermatologists have the ability to guide and motivate patients, such as Ms. A, to make changes that help them increase their healthy lifespan and quality of life as they age.

## Declaration of Competing Interest

None.
